# Associations between breast cancer subtype and neighborhood socioeconomic and racial composition among Black and White women

**DOI:** 10.1007/s10549-020-05545-1

**Published:** 2020-01-30

**Authors:** Erin Linnenbringer, Arline T. Geronimus, Kia L. Davis, John Bound, Libby Ellis, Scarlett L. Gomez

**Affiliations:** 1 Population Studies Center, Institute for Social Research, University of Michigan, Ann Arbor, MI USA; 2 Division of Public Health Sciences, Department of Surgery, Washington University School of Medicine, 660 S. Euclid Ave., Campus Box 8100, St. Louis, MO 63110 USA; 3 Cancer Prevention Institute of California, Fremont, CA USA; 4 London School of Hygiene & Tropical Medicine, London, UK; 5 Department of Epidemiology and Biostatistics, University of California San Francisco, San Francisco, CA USA; 6 Helen Diller Family Comprehensive Cancer Center, University of California, San Francisco, CA USA; 7 Department of Health Behavior & Health Education, University of Michigan School of Public Health, Ann Arbor, MI USA

**Keywords:** Breast cancer subtype, Health inequalities, Neighborhood racial density, Socioeconomic status

## Abstract

**Purpose:**

Studies of Black–White differences in breast cancer subtype often emphasize potential ancestry-associated genetic or lifestyle risk factors without fully considering how the social or economic implications of race in the U.S. may influence risk. We assess whether neighborhood racial composition and/or socioeconomic status are associated with odds of triple-negative breast cancer (TNBC) diagnosis relative to the less-aggressive hormone receptor-positive/HER2-negative subtype (HR+ /HER−), and whether the observed relationships vary across women’s race and age groups.

**Methods:**

We use multilevel generalized estimating equation models to evaluate odds of TNBC vs. HR+ /HER2− subtypes in a population-based cohort of 7291 Black and 74,208 White women diagnosed with breast cancer from 2006 to 2014. Final models include both neighborhood-level variables, adjusting for individual demographics and tumor characteristics.

**Results:**

Relative to the HR+ /HER− subtype, we found modestly lower odds of TNBC subtype among White women with higher neighborhood median household income (statistically significant within the 45–64 age group, OR = 0.981 per $10,000 increase). Among Black women, both higher neighborhood income and higher percentages of Black neighborhood residents were associated with lower odds of TNBC relative to HR+ /HER2−. The largest reduction was observed among Black women diagnosed at age ≥ 65 (OR = 0.938 per $10,000 increase; OR = 0.942 per 10% increase in Black residents).

**Conclusion:**

The relationships between neighborhood composition, neighborhood socioeconomic status, and odds of TNBC differ by race and age. Racially patterned social factors warrant further exploration in breast cancer subtype disparities research.

## Introduction

Disparities between Black and White women are well documented across the breast cancer continuum. Compared to White women, Black women have less access to quality mammography services, experience longer diagnostic and treatment delays, are more likely to receive suboptimal care once treatment is initiated, and are more likely to die of the disease [1–4]. Disparities in several clinical features of breast cancer are also well documented [5, 6], but the unequal distribution of breast cancer subtypes is particularly noteworthy.

Breast cancer subtype is intrinsic to tumor development [7], making it an early and important source of inequality. Relative to White women, Black women with breast cancer are twice as likely to be diagnosed with triple-negative breast cancer (TNBC), defined as tumors that are lacking or express very low levels of estrogen receptors (ER), progesterone receptors (PR), and human epidermal growth factor receptor 2 (HER2) [8–10]. Women with TNBC are more likely to have larger and higher grade tumors, a shorter time to relapse, and lower survival rates than women with ER and/or PR positive (referred to as hormone receptor positive, or HR+), HER2 negative (HER2−) tumors [11–15]. Women from lower socioeconomic areas also have higher rates of TNBC, but the complex relationships among race, socioeconomic status (SES), and breast cancer subtype etiology have not been adequately addressed [16–19].

Racial residential segregation is a structural factor that reinforces the complex relationship between race and SES [20]. The relationship between racial residential segregation and breast cancer disparities is similarly complex, with multiple potential protective and deleterious pathways [18]. For example, racial residential segregation measured at the metropolitan level has been associated with reduced access to adequate breast cancer treatment [21] and increased risk of breast cancer mortality [22, 23]. However, the creation of neighborhoods with a high proportion of one racial group—which is integral to racial residential segregation at the metropolitan level—may also yield underexplored protective resources for racial minority residents living in a majority-minority neighborhoods. Similar to ethnic enclaves, neighborhood-level resources could include greater access to social network resources that could reduce breast cancer risk and improve outcomes, net of the socioeconomic disadvantages that may also be more prevalent within minority-majority neighborhoods [18, 24, 25]. Given the largely atheoretical approach of prior research and the measurement issues associated with interrelationships among metropolitan-level racial segregation, neighborhood-level racial density, individual-level race, and SES, it is unsurprising that the existing breast cancer disparities evidence is decidedly mixed [22, 23, 26–28].

The weathering hypothesis [29] provides an established framework exploring how social, political, and economic marginalization impacts the health of Black women over the life course [25, 30]. Weathering refers to the cumulative biological effects of racially stratified life experiences, exposure to stressors, and access to coping resources that may collectively contribute to population differences in health [31, 32]. For example, findings from the Black Women’s Health Study suggest that living in more integrated neighborhoods compared to segregated neighborhoods may lead to more frequent racial discrimination [33], which has been identified as a potential risk factor for the development of breast cancer among Black women [34]. Given these findings and biological evidence of increased aggressive mammary tumors in socially isolated rodents [24, 25], we hypothesize that Black women with breast cancer who reside in neighborhoods with higher proportions of Black residents will have *lower* odds of TNBC relative to HR+/HER2− disease, net of socioeconomic characteristics. From the weathering perspective, we anticipate that this relationship between racial density and breast cancer subtype will be robust to adjustment for neighborhood socioeconomic status reflective of the role of deeply rooted social ties and dense networks in mitigating the collective distress that activates harmful physiological mechanisms [32].

## Materials and methods

### Study subjects

Cases were drawn from the California Cancer Registry (CCR). Case addresses were geocoded and linked to 17,688 California census block groups included in the California Neighborhoods Data System (CNDS) [35]. A total of 118,225 non-Hispanic White and non-Hispanic Black female residents of California who were diagnosed with a first primary invasive breast cancer at age 18 or older between January 1, 2006 and December 31, 2014 were initially eligible for the study. This period reflects the beginning of widespread HER2 status reporting in 2006 through the most recent available data. Women whose addresses could not be geocoded to a census block group (*n* = 125; 0.1%) or lived in a block group with missing (*n *= 361; 0.3%) or low-quality estimates (i.e., those with high or missing coefficient of variation data) of median household income, (*n *= 7802; 6.6%) were removed from the sample. Of the 109,937 remaining eligible women, 14,172 (12.9%) were missing ER, PR, and/or HER2 data. Older women (mean age 64.3 years vs. 61.7 years; *p* < 0.001), Black women (13.9% vs. 12.8% White; *p *= 0.001), and women who were classified as missing or unknown with regards to insurance status, tumor stage, and tumor grade (*p *< 0.001 for each variable) were significantly more likely to also be missing data on ER, PR, or HER2 status. As the focus of our study was to compare the odds of TNBC versus HR+/HER2− disease, we excluded 14,266 women whose tumors were not classified in either of these subtypes. A total of 81,499 women met the final eligibility criteria and are included in the analysis.

The University of Michigan and the California Health and Human Services Agency’s institutional review boards approved the study.

### Measures

#### Individual-level variables

##### ER/PR/HER2 expression

The CCR has been collecting ER and PR status since 1990, recording either dextran-coated charcoal ER and PR assays or immunohistochemical (IHC) staining results. Reflective of guideline changes, cases diagnosed prior to 2010 were considered negative with < 5% nuclear staining, while those diagnosed from 2010 onward were coded negative with < 1% nuclear staining [36]. HER2 status was assessed by IHC (scores of 0 and 1+  = negative) or fluorescence in situ hybridization (FISH; < 2 copies or HER2 gene = negative). Women with negative results on all three assays were classified as having TNBC, while women with a HER2 negative assay and a positive result on the ER and/or PR assay were assigned the HR+ /HER2− subtype.

##### Race

We examined two mutually exclusive groups, non-Hispanic Whites (hereafter referred to as Whites) and non-Hispanic Blacks (hereafter, Blacks). Race/ethnicity data are primarily derived from patients’ medical records, and have been shown to be of good quality [37, 38].

##### Age

Age at the time of diagnosis is included as a continuous variable in all models. Additionally, age-stratified models were created to assess potential variation in breast cancer subtype odds ratios across the adult lifespan. Informed by the weathering hypothesis and serving as a crude proxy of menopausal status [39, 40], the stratified analyses consist of young pre-menopausal women (< age 45), peri- and post-menopausal women (ages 45–64), and elderly women (ages 65+).

Additional covariates examined include year of diagnosis, marital status (married, single never married, separated, divorced, widowed, unmarried domestic partner, and unknown), payer source at diagnosis (private insurance; uninsured or self-pay; publicly funded (e.g., Medicare, Medicaid, Indian Health Service, or county-funded); military sponsored (e.g., TriCare or Veterans Administration); and unknown), stage (SEER 1977/2000 summary stage categories of local, regional, distant, and unknown), and grade (I, II, III, IV, and unknown).

#### Neighborhood-level variables

##### Neighborhood racial density

Measures of neighborhood racial density are derived from the 2007–2011 American Community Survey and reflect the percentage of non-Hispanic White and non-Hispanic Black residents living within Census-defined block groups of 600 to 3000 residents.

##### Neighborhood-level socioeconomic status

Block group median household income estimated from the 2007–2011 American Community Survey were chosen as the single indicator of neighborhood socioeconomic status to avoid issues of multicollinearity [41].

### Statistical analysis

Descriptive statistics for the full study population and each racial subgroup are reported in Table 1. Statistically significant differences between Black and White women were assessed for each independent variable using unadjusted t tests and chi-square tests. Inter-group differences with *p* values ≤ 0.05 are noted in the tables and highlighted in the results section. Given the significant variation across racial groups, all models are adjusted for individual-level sociodemographic characteristics (age, marital status, insurance status, and race in the non-stratified models) as well as clinical features (year of diagnosis, stage at diagnosis, and tumor grade) in Table 1. The covariates central to the aim of this study were measured at the neighborhood (i.e., block group) level.

**Table 1:** Distribution of sociodemographic, clinical, and neighborhood characteristics of HR+/HER− and triple-negative breast cancer cases, by race, California, 2006–2014

	Total sample (*n *= 81,499)	White cases (*n *= 74,208)	Black cases (*n *= 7291)	*p* values
Age in years, mean (SD)	62.3 (13.3)	62.6 (13.2)	59.1 (13.3)	< 0.001
Age categories, no. (%)				
Less than 45 years old	7220 (8.9)	6232 (8.4)	988 (13.6)	
45 to 65 years old	41,337 (50.7)	37,307 (50.3)	4030 (55.3)	
More than 65 years old	32,942 (40.4)	30,669 (41.3)	2273 (31.2)	
Marital status, no. (%):				
Single, never married	12,459 (15.3)	10,131 (13.7)	2328 (31.9)	
Married	43,826 (53.8)	41,361 (55.7)	2465 (33.8)	< 0.001
Separated	811 (1.0)	662 (0.9)	149 (2.0)	
Divorced	9603 (11.8)	8566 (11.5)	1037 (14.2)	
Widowed	11,654 (14.3)	10,689 (14.4)	965 (13.2)	
Unmarried domestic partner	207 (0.3)	194 (0.3)	13 (0.2)	
Unknown	2939 (3.6)	2605 (3.5)	334 (4.6)	
Primary insurer, No. (%):				
Uninsured/self-pay	493 (0.6)	404 (0.5)	89 (1.2)	
Private	49,101 (60.3)	44,938 (60.6)	4163 (57.1)	< 0.001
Public	29,249 (35.9)	26,438 (35.6)	2811 (38.6)	
Military	529 (0.7)	467 (0.6)	62 (0.9)	
Unknown	2127 (2.6)	1961 (2.6)	166 (2.3)	
Summary stage, No. (%):				
Localized	54,024 (66.3)	49,802 (67.1)	4222 (57.9)	< 0.001
Regional	23,819 (29.2)	21,266 (28.7)	2553 (35.0)	
Remote	3384 (4.2)	2912 (3.9)	472 (6.5)	
Unknown	272 (0.3)	228 (0.3)	44 (0.6)	
Grade, No. (%):				
I; well differentiated	22,203 (27.2)	20,933 (28.2)	1270 (17.4)	< 0.001
II; moderately differentiated	35,286 (43.3)	32,643 (44.0)	2643 (36.3)	
III; poorly differentiated	20,402 (25.0)	17,413 (23.5)	2989 (41.0)	
IV; undifferentiated	540 (0.7)	456 (0.6)	84 (1.2)	
Unknown	3068 (3.8)	2763 (3.7)	305 (4.2)	
Subtype, No. (%)				
HR+ (ER+ and/or PR+)/HER2−	70,347 (86.3)	65,067 (87.7)	5280 (72.4)	< 0.001
Triple negative (ER−/PR−/HER2−)	11,152 (13.7)	9141 (12.3)	2011 (27.6)	
Neighborhood SES, mean (SD)				
BG median household income	$79,908 (36,784)	$81,837 (36,886)	$60,267 (29,214)	< 0.001
Neighborhood racial density				
BG % non-Hispanic White; mean (SD)	57.1 (24.1)	60.3 (22.2)	24.9 (22.3)	< 0.001
BG % non-Hispanic Black; mean (SD)	5.0 (10.0)	3.2 (5.0)	22.6 (22.8)	< 0.001

To test the hypothesized relationships among neighborhood racial density, neighborhood socioeconomic status, and odds of TNBC versus HR+/HER2− subtype, two-level generalized estimating equation (GEE) models were constructed using the XTGEE command in Stata 14. This population average approach accounts for the clustering of individual cases within census block groups even when neighborhood-level variables are not included in the model, but is not subject to the modeling and distribution assumptions that underlie multilevel mixed effects models [42]. Given the large number of clusters (block groups), the relatively small number of cases per cluster (mean = 4.6 cases per block group in the full sample, range 1 to 44) and the conceptual emphasis on the effects of cluster-level predictors, population average models are well-suited to the current research [43]. The odds ratios generated by population average models are interpreted in a similar manner as standard logistic regression models, with the parameter estimates describing the effect of each predictor averaged across all block groups. The two-level population average models were first constructed for the full study sample, then stratified by race. Each of the racial subsamples were further stratified by age group to examine potential variation in the magnitude or direction of associations among younger, middle-aged, and older Black and White women.

## Results

### Descriptive statistics

Table 1 illustrates the unadjusted demographic characteristics of the study sample across all individual- and neighborhood-level variables. Compared to White women, the mean age at diagnosis was significantly lower for Black women (59.1 vs. 62.6 years). Black women in this sample were significantly less likely than White women to be: married (33.8% vs. 55.7%); have private health insurance (57.1% vs. 60.6%); and be diagnosed with an early stage (57.9% vs. 67.1%), low grade (17.4% vs. 28.2%), or HR+/HER2− tumor (72.4% vs. 87.7%).

Each of the three neighborhood-level variables were correlated with one another. Across the 17,477 block groups, median neighborhood household income was positively associated with the percentage of White residents (*r* = 0.44, *p *< 0.01) and negatively associated with the percentage of Black residents (*r* = − 0.25, *p *< 0.01). These block group-level associations were reflected in the individual-level distribution of Black and White women across neighborhoods. As reported in Table 1, the median neighborhood household income for Black women in our sample was significantly lower than that of White women ($81,837 vs. $60,267), while the mean percentage of Black neighborhood residents was much greater than that of White women (22.6% vs. 3.2%).

### Multivariable analyses

Relative to White women, the odds of having TNBC versus HR+/HER2− breast cancer was 2.60 times higher for Black women in the baseline model (Table 2, Model 1; 95% CI 2.46–2.75). Adjusting for individual-level sociodemographic characteristics and clinical characteristics reduced the OR for TNBC among Black women (Model 3; OR = 1.95, 95% CI 1.82–2.09). The addition of neighborhood-level median household income resulted in a further reduction in the OR for TNBC among Black women (Model 4; OR = 1.90, 95% CI 1.77–2.04). In this fully adjusted model, every $10,000 USD increase in block group median household income was associated with a statistically significant 1.4% decrease in the odds of TNBC relative to HR+/HER2− breast cancer (Model 4; OR = 0.986, 95% CI 0.98–0.99).

**Table 2:** Adjusted odds ratios (95% confidence intervals) for TNBC vs. HR+ /HER2− subtype, by individual-level sociodemographic characteristics, tumor characteristics, and block group-level median household income, California Cancer Registry 2006–2014

	Triple negative vs. HR+/HER2− subtype			
	Model 1	Model 2	Model 3	Model 4
	OR (95% CI)	OR (95% CI)	OR (95% CI)	OR (95% CI)
Individual level				
Year of diagnosis	0.96 (0.95–0.96)*	0.96 (0.95–0.96)*	0.96 (0.95–0.97)*	0.96 (0.95–0.97)*
Age at diagnosis (in years.)	0.98 (0.98–0.99)*	0.98 (0.98–0.98)*	0.99 (0.99–1.00)*	0.99 (0.99–1.00)*
Race				
White (ref.)	1.00	1.00	1.00	1.00
Black	2.60 (2.46–2.75)*	2.59 (2.44–2.74)*	1.95 (1.82–2.09)*	1.90 (1.77–2.04)*
Marital status				
Married (ref.)		1.00	1.00	1.00
Never married		0.94 (0.89–1.00)^	0.89 (0.83–0.95)*	0.88 (0.82–0.94)*
Separated		0.89 (0.73–1.09)	0.97 (0.78–1.21)	0.95 (0.76–1.19)
Divorced		1.05 (0.98–1.12)	1.02 (0.95–1.10)	1.01 (0.94–1.08)
Widowed		1.08 (1.01–1.16)^	0.98 (0.90–1.06)	0.96 (0.89–1.04)
Unmarried partner		0.82 (0.52–1.27)	0.93 (0.58–1.51)	0.92 (0.57–1.48)
Unknown		1.12 (0.95–1.24)^	1.10 (0.98–1.24)	1.08 (0.96–1.22)
Primary payer				
Private insurance (ref.)		1.00	1.00	1.00
Not insured		1.33 (1.06–1.68)^	1.15 (0.89–1.48)	1.14 (0.88–1.47)
Public		1.12 (1.07–1.18)*	1.02 (0.97–1.08)	1.01 (0.96–1.07)
Military		1.01 (0.79–1.31)	0.96 (0.72–1.27)	0.94 (0.71–1.25)
Unknown		1.22 (1.08–1.37)*	1.19 (1.04–1.37)^^^	1.18 (1.03–1.35)^^^
Summary stage				
Local (ref.)			1.00	1.00
Regional			0.77 (0.74–0.81)*	0.77 (0.74–0.83)*
Remote			0.83 (0.75–0.92)*	0.83 (0.74–0.92)*
Unknown/NOS			1.09 (0.77–1.54)	1.08 (0.76–1.53)
Tumor grade				
I (ref.)			1.00	1.00
II			4.59 (4.05–5.21)*	4.59 (4.04–5.20)*
III			50.54 (44.74–57.09)*	50.37 (44.59–56.90)*
IV			60.90 (49.27–75.29)*	60.54 (48.97–74.85)*
Unknown/NOS			16.01 (13.73–18.67)*	15.94 (13.67–18.60)*
Block group level				
Median HH income^a^				0.99 (0.98–0.99)*

^a^Median household income; units = $10,000 (in 2009 U.S. dollars) Models are adjusted for all variables listed

^^^*p* value ≤ 0.05

*p value ≤ 0.01

Different patterns emerged across neighborhood-level variables when the full study sample was stratified by race (Table 3). Within the White subpopulation, the relationship between block group median household income and odds of TNBC vs. HR+/HER2− breast cancer was virtually identical to the relationship seen in the full population (Model 4; OR = 0.99, 95% CI 0.98–0.99). The block group percentage of White residents was not significantly associated with odds of TNBC vs. HR+/HER2− breast cancer among White women (Model 6A; OR = 1.00, 95% CI 0.99–1.01), and did not alter the magnitude or direction of the relationship between block group median household income and TNBC when added to the model.

**Table 3:** Total study population and race-stratified adjusted odds ratios (95% confidence intervals) for TNBC vs. HR+ /HER2− subtype, by neighborhood-level median household income & racial density, California Cancer Registry, 2006–2014

	Triple negative vs. HR+/HER2− subtype				
	Model 4	Model 5A	Model 5B	Model 6A	Model 6B
	OR (95% CI)	OR (95% CI)	OR (95% CI)	OR (95% CI)	OR (95% CI)
Total sample (*n *= 81,499)					
Block group median HH income^a^	0.99 (0.98–0.99)*			0.98 (0.98–0.99)*	0.98 (0.98–0.99)*
Block group % White^b^		1.00 (0.99–1.01)		1.00 (0.99–1.01)	
Block group % Black^c^			0.98 (0.96–1.01)		0.97 (0.95–1.00)^^^
Stratifed analysis: White women only					
Block group median HH income	0.99 (0.98–0.99)			0.99 (0.98–0.99)	
Block group % White		0.99 (0.98–1.00)		1.00 (0.99–1.01)	
Stratifed analysis: Black women only					
Block group median HH income	0.98 (0.96–1.00)				0.97 (0.95–1.00)^^^
Block group % Black			0.96 (0.94–0.99)*		0.96 (0.93–0.98)*

Adjusted for all covariates included in Table 2 models: age at diagnosis, year of diagnosis, marital status, insurance status, stage at diagnosis, and tumor grade

^a^Block group median household income; units = $10,000 (in 2009 U.S. dollars)

^b^Units = 10-point change in percent of White block group residents

^c^Units = 10-point change in percent of Black block group residents

^^^*p* value ≤ 0.05

*p value ≤ 0.01

Block group median household income had a similar OR for TNBC vs. HR+/HER2 breast cancer within the Black subpopulation, approaching statistical significance (Model 4; OR = 0.98; 95% CI 0.96–1.00). However, unlike the White subgroup, the percentage of same-race neighborhood residents was significantly associated with lower odds of TNBC vs. HR+/HER2− breast cancer among Black women, both when included as the only neighborhood-level variable (Model 5B; OR = 0.96, 95% CI 0.94–0.99) and when adjusting for neighborhood median household income (Model 6B; OR = 0.96, 95% CI 0.93–0.98). Adjusting for all individual- and neighborhood-level variables, we found that a 10% increase in the proportion of Black neighborhood residents was associated with a 4.1% decrease in odds of TNBC diagnosis, relative to the HR+/HER2− subtype.

The patterns observed in race-specific subpopulations also varied across age groups (Table 4). Within the White subpopulation, increasing neighborhood median household income was associated with significantly lower odds of TNBC vs. HR+/HER2− breast cancer among women ages 45 to 65 (OR = 0.98, 95% CI 0.97–0.99). No other significant relationships between breast cancer subtype odds and either of the neighborhood-level variables were identified in the other two White age groups.

**Table 4:** Age-stratified, race-specific adjusted odds ratios (and 95% confidence intervals) for TNBC vs. HR+/Her2− subtype by neighborhood-level median household income and racial density, California Cancer Registry, 2006–2014

	Triple negative vs. HR+/HER2− subtype		
	Model 7	Model 8	Model 9
	OR (95% CI)	OR (95% CI)	OR (95% CI)
Age-stratifed analyses: White women only			
Age at diagnosis < 45 years			
Neighborhood median HH income^a^	0.98 (0.97–1.00)		0.99 (0.97–1.01)
Neighborhood % White^b^		0.98 (0.95–1.01)	0.99 (0.95–1.02)
Age at diagnosis = 45 to 64 years			
Neighborhood median HH income	0.98 (0.97–0.99)*		0.98 (0.97–0.99)
Neighborhood % White		0.99 (0.97–1.01)	1.00 (0.98–1.02)
Age at diagnosis ≥ 65 years			
Neighborhood median HH income	0.99 (0.98–1.01)		0.99 (0.98–1.01)
Neighborhood % White		1.00 (0.98–1.02)	1.00 (0.98–1.02)
Age-stratifed analysis: Black women only			
Age at dx < 45 years			
Neighborhood median HH income	0.99 (0.94–1.05)		0.99 (0.93–1.04)
Neighborhood % Black^c^		0.98 (0.91–1.05)	0.97 (0.90–1.05)
Age at dx = 45 to 64 years			
Neighborhood median HH income	0.99 (0.97–1.02)		0.99 (0.96–1.02)
Neighborhood % Black		0.97 (0.94–1.00)†	0.97 (0.93–1.00)†
Age at dx ≥ 65 years			
Neighborhood median HH income	0.94 (0.91–0.98)*		0.94 (0.90–0.98)*
Neighborhood % Black		0.95 (0.91–1.00)^	0.94 (0.90–0.99)^

Adjusted for all covariates included in Table 2 models: age at diagnosis, year of diagnosis, marital status, insurance status, stage at diagnosis, and tumor grade

^a^Median household income; units = $10,000 (2009 U.S. dollars)

^b^Units = 10-point change in percent of White block group residents

^c^Units = 10-point change in percent of Black block group residents

^†^*p* value ≤ 0.10

^^^*p* value ≤ 0.05

*p value ≤ 0.01

Conversely, Black women who were diagnosed with breast cancer at age 65 or older had 6.2% lower odds of TNBC vs. HR+/HER2− breast cancer with a $10,000 unit increase in block group median household income (Model 9; OR = 0.94, 95% CI 0.90–0.98), and a 5.8% lower odds of TNBC vs. HR+/HER2− breast cancer with a 10% unit increase in block group percentage Black (OR = 0.94, 95% CI 0.90–0.99). A trend toward lower odds of TNBC with increasing block group percentage Black (OR = 0.97; 95% CI 0.93–1.00) was observed among Black women ages 45 to 64. Neither the neighborhood SES nor neighborhood racial composition was significantly associated with odds of TNBC vs. HR+/HER2− breast cancer among the under 45 age group, although the point estimates suggest similar relationships may exist in the hypothesized direction.

## Discussion

Our population-based study of California women indicates that, among Black women diagnosed with breast cancer, living in neighborhoods with greater proportions of Black residents is associated with significantly lower odds of being diagnosed with TNBC relative to the HR+/HER2− subtype. No significant changes in odds of TNBC were observed among White women living within neighborhoods with greater proportion of White residents. As noted earlier, Warner & Gomez found similar relationships between racial density and breast cancer stage at diagnosis and mortality among Black women [28], but to our knowledge, this is the first study to identify an association between neighborhood racial density and odds of specific breast cancer subtypes.

We also found that, for both Black and White women with breast cancer, living in areas with higher neighborhood median household incomes was associated with lower odds of TNBC relative to the HR+/HER− subtype. These results are consistent with prior research on breast cancer subtype distribution across area-based SES levels [19, 44, 45]. Importantly, our findings regarding neighborhood racial composition and odds of TNBC among Black women with breast cancer remain statistically significant after accounting for neighborhood median household income. The association between odds of TNBC and each neighborhood factor was strongest among Black women ≥ 65 years old, pointing toward opportunities for future study and intervention.

To our knowledge, only one study has examined the relationship between a well-defined measure of residential segregation and breast cancer subtype. Krieger et al. [46] developed county-level measures of the Index of Concentration at the Extremes (ICE) for economic, racial, and racialized economic segregation and then assessed the odds of ER+ versus ER− breast cancer using the SEER 13 (US Surveillance, Epidemiology, and End Results) data set. Like our findings on neighborhood SES, they found that women residing in counties with a greater concentration of high-income residents had significantly higher odds of the more clinically favorable ER+ breast cancer subtype. Unlike our results, they found counties with greater concentrations of non-Hispanic White residents relative to Black residents also had higher odds of the more clinically favorable ER+ breast cancer subtype, but statistical significance was only reached when comparing the this highest vs. lowest quintile (OR = 1.27, 95% CI 1.11–1.45). Notably, race-stratified analyses were not conducted and thus it is unclear whether the observed relationship between racial segregation and ER status holds true within the population of Black women with breast cancer or at the neighborhood level.

The health impact of racial segregation is clearly complex, but theoretical frameworks can help disentangle competing risks [47]. The weathering hypothesis points to factors that might mitigate the disadvantages associated with racial residential segregation and contribute to the associations we have identified. Specifically, for Black women, the benefits of living in neighborhoods with greater proportion of Black residents may include: reduced exposure to interpersonal racial discrimination [33, 48], greater access to protective cultural frameworks [49], and/or greater opportunities to develop deeply rooted social ties, strategies for economic risk pooling, and other forms of social support [50, 51]. These benefits may help compensate for the social and economic disadvantage that Black women frequently experience.

Investigating the potential link between neighborhood racial concentration and perceived or anticipated racial discrimination is an especially important and currently understudied area of breast cancer subtype disparities research. In one of the very few related studies conducted to date, Taylor and colleagues found that more frequent experiences of everyday discrimination were associated with an increased risk of incident breast cancer among Black Women’s Health Study participants, particularly those under the age of 50 [34]. However, they did not separate breast cancer cases by subtype, so it is unclear as to whether the observed age stratification is due to age-specific variation in the risk associated with discrimination, or if the results are confounded by the well-established increased risk of TNBC among younger women [10]. Innovative work by Krieger et al. did not directly measure interpersonal discrimination, but rather structural racism via the legal codification of racial discrimination in states with Jim Crow laws. They found that Black women with breast cancer who were born in a Jim Crow state prior to the passage of the US Civil Rights Act of 1964 had significantly higher odds of ER- subtype relative to Black women born in other states [52]. Importantly, there was no such geographic difference in breast cancer subtype odds ratios among White women of any age. Taken together and in the context of our study, these findings suggest that exposure to structural racism and/or interpersonal racial discrimination may be associated with increased odds of aggressive breast subtypes among Black women with breast cancer.

Several data limitations are worth noting. First, cases excluded due to missing ER, PR, or HER2 status could introduce selection bias into study sample [53], although simulation studies suggest that regression analyses that adjust for variables associated with missing outcome data produce comparable results to analyses including imputed outcome data [54]. Second, while the importance of the duration and timing of neighborhood-level exposures to the development of specific breast cancer subtypes is not known, it is plausible that using a single time point (e.g., residence at the time of diagnosis) may not adequately capture the association between neighborhood-level characteristics and breast cancer subtype. Cohort studies that include geocoded residential history information are needed to adequately address these issues. Finally, the lack of individual-level biopsychosocial data and breast cancer risk factors (e.g., parity [55]) are potential sources of unobserved heterogeneity. Population-based cancer registries including the CCR do not typically collect this type of data, thereby preventing the analysis of potential pathways linking individual- and neighborhood-level factors to breast cancer subtypes. Despite these limitations, this study makes an important contribution to the breast cancer disparities literature: social-structural factors are associated with odds of TNBC subtypes among both Black and White women with breast cancer, but these associations vary between racial and age groups.

In conclusion, we found that living in neighborhoods with greater proportions of same-race residents is associated with significantly lower odds of being diagnosed with TNBC among Black women but not for White women. This first-known study examining the relationship between neighborhood racial density and odds of specific breast cancer subtypes points to structural factors as potentially significant elements associated with racial inequalities in breast cancer subtypes. Most studies of Black–White differences in breast cancer subtypes focus on ancestry-associated genetic or lifestyle risk factors without fully considering the social construction of race in the U.S. and how it impacts social and physical environmental exposures and protective factors at the population level. As a result, these studies are insufficient in describing the complex and dynamic relationships among individuals, their environment, and breast cancer subtypes. Lastly, disparities in incidence of TNBC are not narrowing. To identify areas of intervention to reduce racial inequalities in breast cancer subtypes, additional research should explore racially patterned physical and social environments that might also pattern breast cancer risk.

## Data Availability

The data that support the findings of this study are available from the California Cancer Registry (CCR) but restrictions apply to the availability of these data, which were obtained following CCR review and approval. Summary data are available from the authors upon reasonable request and with the permission of the CCR. Investigators may also request data directly from the CCR’s website at https://www.ccrcal.org/retrieve-data/data-for-researchers/.
